# Back to basics: tackling the challenges to bedside teaching

**DOI:** 10.2147/AMEP.S74592

**Published:** 2014-12-23

**Authors:** Rizwan Dewji, Abbas Dewji, Dushyanth Gnanappiragasam

**Affiliations:** Imperial College School of Medicine, Imperial College London, London, UK

**Dear Editor**

In recent times, there has been a declining trend in bedside teaching as part of the medical teaching curriculum.^1^ It is clear that this fundamental issue presents a potential barrier to the development of both current and future generations of doctors. Agreeable explanations for the decline in bedside teaching include a more rapid patient turnover, increased reliance on technology in the diagnostic process, and the limited availability of clinician time.

In addition to the aforementioned, it is important to consider other contributing factors, such as the increased availability and use of patient simulators. Some studies have even suggested that use of simulators should be prioritized ahead of bedside teaching for “struggling students” as a way of increasing confidence, proficiency, and long-term patient care.^2^ Alternatively, resource and time constraints may provide an explanation for the reduced amount of bedside teaching received by medical students.^3^ With health care budgets being widely cut and numerous targets being introduced, it is difficult for medical professionals to balance these external pressures and at the same time provide clinical opportunities for future doctors. An example of such a restriction is the National Health Services’ “A&E four-hour target”, which has left minimal time for bedside teaching opportunities. With severe financial sanctions arising from missing one’s targets, it is understandable that doctors are having to prioritize short-term clinical care over bedside teaching in such settings.

Moreover, in endeavoring to overcome the decline in bedside teaching, it may be appropriate to implement innovative strategies. One possible suggestion is to introduce clinical examinations earlier in medical school programs, hence increasing the pressure on students and teachers alike to gain and provide clinical opportunities, respectively. Whilst some universities have clinical examinations throughout medical school training, a significant number do not have clinical examinations or provide clinical experience for the first 2 years of study. As part of the wider strategy, there should be incentives and award schemes for clinicians who participate in bedside teaching, and at the grass root level more opportunities should be provided for trainees and senior students to get involved in teaching bedside skills to junior students. Finally, a shift towards regular assessment of observed history and clinical examinations of “real patients” in both the inpatient and outpatient settings should be implemented as part of each clinical rotation in the medical curriculum.

Currently we are experiencing a dynamic change in the medical curriculum, as there is a shift from traditional bedside teaching to more technology-focused clinical learning tools. In conclusion, we believe bedside teaching should be preserved at any cost and should form the core of clinical teaching. However, the role of technology should not be underestimated and where appropriate, online and mobile virtual tools should be utilized to supplement and thus enhance each student’s learning experience.
